# Rational design of a NIR fluorescent probe for highly selective sensing of NAD(P)H quinone oxidoreductase 1 in living systems

**DOI:** 10.3389/fchem.2025.1626741

**Published:** 2025-06-24

**Authors:** Fangyu Yang, Li Zhu, Fei Yan, Houli Zhang, Guobiao Liang

**Affiliations:** ^1^ Department of Neurosurgery, General Hospital of Northern Theater Command, Shenyang, China; ^2^ Second Affiliated Hospital, Dalian Medical University, Dalian, China; ^3^ College of pharmacy, Dalian Medical University, Dalian, China

**Keywords:** NQO1, fluorescent probe, fluorescence imaging, near-infrared, tumor

## Abstract

NAD(P)H quinone oxidoreductase 1 (NQO1) plays a critical role in catalyzing the reduction of quinones, thereby exerting both chemical protection and biological activation. The expression level of NQO1 in various tumor cells, especially glioma, lung cancer, colon cancer and breast cancer cells, is higher than that in normal tissues, the detection of NQO1 activity is important to understand tumor development mechanisms. Herein, a near-infrared (NIR) fluorescent probe DDANQ was rationally designed and synthesized through a systematic approach. The optical properties and enzyme selectivity of DDANQ were also elucidated by chemical calculation and molecular docking methods. The results showed that DDANQ showed excellent stability, and displayed good selectivity and sensitivity toward NQO1. DDANQ could be used as a molecular tool to elucidate the activity and function of NQO1 in physiological and pathological processes, which could provide valuable insights for tumor diagnosis and treatment.

## 1 Introduction

NAD(P)H quinone oxidoreductase 1 (NQO1) also known as DT-diaphorase, is a flavin protease that participates in the reduction reaction of quinone. NQO1 contributes to cellular defense and metabolic activation of quinones, responsible for the detoxification of exogenous drugs (Siegel et al., 2012b), and it could also metabolize a variety of benzene chemical carcinogens in the human body (Siegel et al., 2012a). NQO1 is expressed mainly in the cytoplasm of mammals, but less expressed in the nucleus. In contrast, the expression of NQO1 in a variety of tumor cells is much higher, especially glioma, lung cancer, colon cancer and breast cancer cells, than that in normal tissues cells (Bey et al., 2007; Yang et al., 2014; Li et al., 2015; Lei et al., 2020; Jiang et al., 2024). Thus, the detection of NQO1 activity is important to understand the development of tumors. In the present work, the detection methods for NQO1 cover multiple levels such as the gene level, protein level and functional activity detection. The polymorphism of the NQO1 gene was detected by PCR-RFLP (polymerase chain reaction - Restriction Fragment Length polymorphism) technology (Yan et al., 2022). The NQO1 protein in tissues, serum, plasma or cell supernatants was detected by immunohistochemistry (Qiu et al., 2023) or Western blotting (Zhu et al., 2023). In terms of functional activity detection, fluorescent probe technologies such as two-photon fluorescent probes have been developed (Jiang et al., 2024).

Fluorescence technology is diffusely applied in many aspects of life science because of its characteristics of high sensitivity, high selectivity and non-invasive imaging (Sargazi et al., 2022). Although some fluorescent probes have been exploited for the monitor of NQO1 (Silvers et al., 2013; Liu et al., 2017; Son et al., 2019; Yao et al., 2020; Wang et al., 2021; Guisan-Ceinos et al., 2022; Wang et al., 2022), fluorescent probes with good chemical stability for the detection of NQO1 are still needed (Lei et al., 2017; Mao et al., 2019; Yang et al., 2021; Bai et al., 2022; Han et al., 2022; Wang et al., 2022). Near-infrared (NIR) fluorescent probes are protected from the interference of signals from the biological background, and NIR light has less light damage, less light scattering and deeper tissue imaging penetration (Owens et al., 2016; Montecinos-Franjola et al., 2020).

Herein, a NIR fluorescent probe DDANQ (*N*-(6,8-dichloro-9,9-dimethyl-7-oxo-7,9-dihydroacridin-2-yl)-3-methyl-3-(2,4,5-trimethyl-3,6-dioxocyclohexa-1,4-dien-1-yl)butanamide) was reasonably engineered and developed to detect the activity of NQO1, which displayed excellent selectivity and sensitivity toward NQO1. Based on its good optical imaging properties, DDANQ was further used in living systems, including cells and tissues. It has been proved that the probe has reliable diagnostic performance for imaging of cancer biomarkers, and could expressly detect the endogenous NQO1 activity in cancer tissues, and has great potential in intraoperative navigation.

## 2 Materials and methods

### 2.1 Materials

CYP1A2, CYP3A4 and CYP2E1 were obtained from BD Biosciences (USA). Monoamine Oxidase A (MAO-A), Monoamine Oxidase B (MAO-B) and flacin-containing monooxygenase 3 (FMO3) were purchased from Corning Incorporated (USA). NAD(P)H quinone oxidoreductase 1 (NQO1) and Nitroreductase (NTR) were purchased from Sigma-Aldrich (Merck, USA). Alcohol dehydrogenase (ADH) and Acet-aldehyde dehydrogenase (ALDH) were purchased from Shanghai Yuanye (Shanghai, China). Chemicals reagents for the synthesis of fluorescent probe and fine chemicals were produced by J&K Scientific (Beijing, China). The A549 cells, MDA-MB-231 cells and Hela cells were purchased from ATCC (Manassas, VA).

### 2.2 The synthesis of candidate fluorescent molecules for the detection of NQO1 activity

According to the metabolic characteristics of NQO1, DDAO (1,3-dichloro-7-hydroxy-9,9-dimethylacridin-2(9*H*)-one) and DDAN (7-amino-1,3-dichloro-9,9-dimethylacridin-2(9*H*)-one) with excellent fluorescence properties were selected as fluorophores, and benzoquinone was introduced as the recognition site, DDAOQ (6,8-dichloro-9,9-dimethyl-7-oxo-7,9-dihydroacridin-2-yl 3-methyl-3-(2,4,5-trimethyl-3,6-dioxocyclohexa-1,4-dien-1-yl)butanoate) and DDANQ are synthesized as the fluorescent candidate molecules for the detection of NQO1 activity.

DDAO (0.05 mmol), MEDB (0.05 mmol), DMAP (0.1 mmol), and EDC·HCl (0.5 mmol) were dissolved in CH_2_Cl_2_, the solution was stirred overnight at room temperature, and the reaction mixture was evaporated under reduced pressure. Then the crude product was purified by silica gel chromatography (EtOAc: hexane = 5:1, v: v) to afford 19.5 mg of DDAOQ. ^1^H NMR (600 MHz, CDCl_3_) *δ* 7.63 (d, *J* = 7.0 Hz, 2H), 7.12 (d, *J* = 2.3 Hz, 1H), 7.03 (dd, *J* = 8.5, 2.3 Hz, 1H), 3.29 (s, 2H), 2.20 (s, 3H), 1.95 (d, *J* = 3.2 Hz, 6H), 1.85 (s, 6H), 1.56 (s, 2H), 1.54 (s, 6H). ^13^C NMR (150 MHz, CDCl_3_) *δ* 190.81, 187.35, 173.16, 170.83, 153.09, 151.40, 149.84, 142.66, 139.67, 139.42, 138.90, 138.57, 137.41, 135.62, 133.07, 121.47, 119.69, 47.77, 39.04, 38.40, 29.05, 26.66, 14.49, 12.67, 12.20. High-resolution mass spectrum (ESI positive) calcd for [M + H]^+^ 540.1339, found 540.1316.

DDAN (0.05 mmol), MEDB (0.05 mmol), DMAP (0.1 mmol), and EDC·HCl (0.5 mmol) were dissolved in CH_2_Cl_2_, the solution was stirred overnight at room temperature, and the reaction mixture was evaporated under reduced pressure. Then the crude product was purified by silica gel chromatography (CH_2_Cl_2_: hexane = 5:1, v: v) to afford 10.2 mg of DDANQ. ^1^H NMR (600 MHz, CDCl_3_) *δ* 7.76 (d, *J* = 2.0 Hz, 1H), 7.62 (s, 1H), 7.57 (d, *J* = 8.5 Hz, 1H), 7.35 (dd, *J* = 8.4, 2.3 Hz, 2H), 3.09 (s, 2H), 2.18 (s, 3H), 1.98 (s, 3H), 1.94 (s, 3H), 1.86 (s, 6H), 1.52 (s, 6H). ^13^C NMR (150 MHz, CDCl_3_) *δ* 191.76, 187.38, 173.22, 170.64, 152.31, 148.71, 142.98, 141.30, 140.73, 139.81, 139.41, 138.98, 138.79, 137.60, 136.97, 135.03, 133.05, 118.68, 116.95, 50.79, 39.13, 29.26, 26.74, 14.32, 12.64, 12.22. High-resolution mass spectrum (ESI negative) calcd for [M-H]^-^ 537.1353, found 537.1375.

### 2.3 The incubation conditions

The standard incubation system consisted of 100 mM potassium phosphate buffer (pH = 7.4), 50 μM FAD (flavin adenine dinucleotide), 200 μM NADH, NQO1 and 10 μM DDANQ, the total volume is 200 μL, in which the proportion of organic regents does not exceed 1% (v/v). The incubation mixture was initiated by adding NADH and incubated at 37°C for 30 min, the incubation was terminated by adding 100 μL acetonitrile and centrifuged at 4°C, 20,000 *g* for 10 min. The supernatants were used for subsequent analysis.

The stability and reaction rate of the two candidate molecules were investigated. According to the above standard incubation system, the concentration of DDAOQ and DDANQ was 10 μM. The final concentrations of NQO1 were 0.125 U/mL. To investigate the stability of DDAOQ and DDANQ, they were incubated with 200 μM NADH at 37°C for 30 min, respectively.

### 2.4 Screening the selectivity of DDANQ

To check the selective sensing capabilities of DDANQ, a series of oxidoreductases including CYP1A2, CYP3A4, CYP2E1, MAO-A, MAO-B, FMO3, NTR, ADH, ALDH, NQO1 were tested in the incubation system mentioned above. Other conditions are as follows, the final concentration of NQO1 was 0.0625 U/mL, other oxidoreductases were 2 μg/mL. One step further, the fluorescence stability of DDANQ with some endogenous and exogenous substances, such as tryptophan (Try), serine (Ser), glutamine (Gln), lysine (Lys), glutathione (Glu), glycine (Gly), arginine (Arg), cysteine (Cys), tyrosine (Tyr), Myristic acid, K^+^, Na^+^, Mg^2+^, Ca^2+^, Ba^2+^, Zn^2+^, Cu^2+^, Ni^2+^, Sn^4+^, Mn^2+^, CO_3_^2-^, SO_4_^2-^, Fe^2+^, Fe^3+^, Cl^−^, NO_2_^−^, HPO_4_^2-^, H_2_PO_4_^−^, the final concentrations were 200 μM.

### 2.5 The molecular docking simulations

The interaction mechanism between probe DDANQ and the NOQ1 protein was investigated. Docking studies were conducted using VINA1.1.2 software to predict the binding energy between the receptor (NOQ1 protein) and the ligand (probe DDANQ) using a semi-empirical free energy model.

### 2.6 The underlying mechanism for the fluorescence properties of DDANQ and DDAN

The geometry of the compound was optimized by using density functional theory (DFT) and time-dependent density functional theory (TD-DFT) at the TPSSh functional in conjunction with the 6-31G (d) basis set. The vibration frequencies analyses were performed for the optimized geometries to identify extreme points (zero imaginary frequencies) located on the respective potential energy surfaces. The Frontier molecular orbital energy levels and their distributions of the highest occupied molecular orbital (HOMO) and the lowest unoccupied molecular orbital (LUMO) were calculated based on the optimized structures. All these calculations were achieved by Gaussian 16 package.

### 2.7 The cells culture and fluorescence imaging

The cell viability of DDANQ was tested by Cell Counting Kit-8 (CCK-8). In brief, A549 cells were cultured in Dulbecco’s Modified Eagle Medium/Nutrient Mixture F-12 with 10% Fetal Bovine Serum (FBS) and 1% penicillin and streptomycin at 37°C in a 5% CO_2_ incubator. A549 cells were seeded at a concentration of 8 × 10^4^/mL in 96-well plate. After the cells were attached to the plate, incubated with different concentrations of DDANQ for 12 h. The medium with DDANQ was then discarded and replaced with 10% (*v/v*) CCK-8 in culture medium. After incubation in an incubator for 1 h, cells were tested the absorbance at 450 nm. In the absence of DDANQ, the cell survival rate was considered as 100%.

Before the cells were seeded, the confocal cell culture dishes were sterilized at first and then cultured to cell adhesion. The inhibitor-treated group was incubated with *β*-lapachone (20 μM) for 30 min, then, the culture medium was replaced with/without 10 μM of DDANQ and incubated at 37°C for another 30 min. After 30 min, the adherent cells were washed with PBS gently and fixed in 4% paraformaldehyde solution in the dark. At last, the samples were imaged under a confocal fluorescence microscope (Leica TCS SP8), the excitation wavelength was set at 633 nm and the collected wavelength range was set as 645–690 nm.

### 2.8 The flow cytometric analysis of NQO1 in cells

The cells were resuspended in PBS and divided into several groups, the volume was 500 μL and the concentration of DDANQ was 10 μM, equal volume of organic solvent was added to the blank group, and the proportion of organic solvent did not exceed 0.5%. The cells were incubated in a 37°C incubator for 15 min, then centrifuged and collected. The residual probe was washed twice by PBS, and the cell suspension was filtered by 0.22 μm and then detected by flow cytometry.

### 2.9 The fluorescence imaging of NQO1 in Hela tumor tissue slices

This study was conducted in strict compliance with the ethical guidelines and protocols sanctioned by the Ethics Committee of Dalian Medical University, ensuring adherence to the highest standards of animal welfare and ethical research practices (ethical approval code: AEE23063). Hela cells were cultured in 1640 with 10% FBS and 1% penicillin and streptomycin. 1 × 10^7^ Hela cells (suspended in 300 μL sterile PBS) were subcutaneously administered into the 5–6 weeks old nude mice to obtain tumor models after 20 days. Whole blood was collected from nude mice after anesthetized with 2.5% avertin (intraperitoneal injection) and the tumor nodules of mice were removed after sacrifice. Then the tumor nodules were soaked in OCT embedding agent, and placed in −80°C refrigerator to let it freeze quickly into chunks. The frozen tumor nodules were put into pre-cooled frozen cryostat microtome (LEICA CM1850) and cut into slices with a thickness of 10 μm.

The slices of blank group and control group were immersed in phosphate buffer (100 mM KH_2_PO4-K_2_HPO_4_, pH 7.4), and the inhibitor group was immersed in phosphate buffer containing 20 μM inhibitor, and pre-incubated in 37°C water bath for 30 min. Phosphate buffer containing cofactors (FAD 50 μM, NADH 200 μM) was dropped to the blank group, and phosphate buffer containing DDANQ 10 μM and cofactors was dropped to the control group. In the inhibitor group, phosphate buffer containing DDANQ 10 μM, cofactors and inhibitor 20 μM was dropped to the slices and incubated in 37°C water bath for 30 min.

After the reaction, the slices were washed with PBS. Then DAPI solution was added for counterstaining, incubated at room temperature (RT) for 10 min. After three times washes, the samples were fixed with 4% paraformaldehyde and sealed with anti-fluorescence quenching sealing solution. The samples were examined under a confocal fluorescence microscope (Leica TCS SP8) (*λ*_ex_ = 633 nm, *λ*_em_ = 645–690 nm).

### 2.10 The fluorescence imaging of NQO1 in human breast cancer slices

For the experiment of fluorescence imaging in human breast cancer slices, the breast cancer specimens were cut to a thickness of 10 μm by a cryostat microtome. At first, the slices were pre-incubated with/without *β*-lapachone (20 μM) for 30 min at 37 °C. Secondly, the slices were incubated with DDANQ (20 μM) for 1 h at 37°C. Thirdly, the slices washed with PBS gently to remove the excess fluorescent probe, then the samples were fixed with 4% polyformaldehyde solution in the dark. Subsequently, after washing with PBS, the samples were dyed with DAPI at RT. Finally, the samples were sealed by anti-fluorescence quenching sealant and were analyzed by confocal fluorescence microscope (*λ*_ex_ = 633 nm, *λ*_em_ = 645–690 nm).

## 3 Results

### 3.1 Design of fluorescent probe and NQO1 response

**SCHEME 1:**
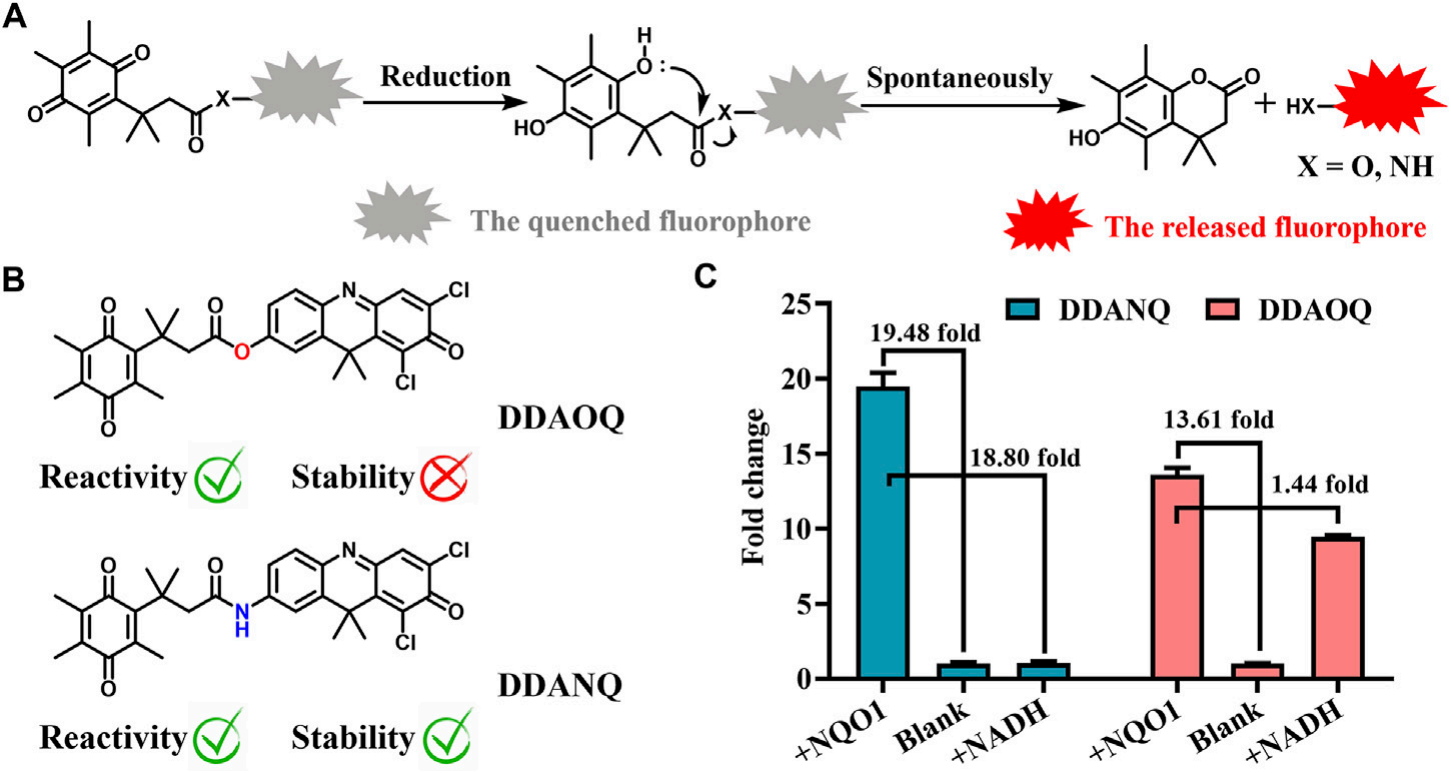
**(A)** Schematic diagram of the catalysis process mediated by NQO1. **(B)** Structures of DDAOQ and DDANQ. **(C)** The sensitivity and stability of DDAOQ and DDANQ.

NQO1 catalyzes the reduction reaction of quinone, thus, benzoquinone was introduced as the recognition site. Then DDAO and DDAN were selected as fluorophores for their excellent optical properties. As can be seen in Scheme 1, both DDAOQ and DDANQ were designed and developed, the synthesis methods are described in the Materials and Methods (Supplementary Schemes S1, S2), and Supplementary Figures S1–S6 showed the structure characterization data. Firstly, the chemical stability was tested, although both DDAOQ and DDANQ could be catalyzed by NQO1, NADH is required to provide hydrogen ions in the catalytic metabolism progress of NQO1, DDAOQ was unstable in the presence of NADH without NQO1, while DDANQ displayed good stably in the present of NADH, as we all known, amide bonds are more stable than ester bonds, thus, DDANQ was selected as the fluorescent probe for the further experiments.

### 3.2 The optical properties of DDANQ toward NQO1 and molecular orbital calculation

**FIGURE 1:**
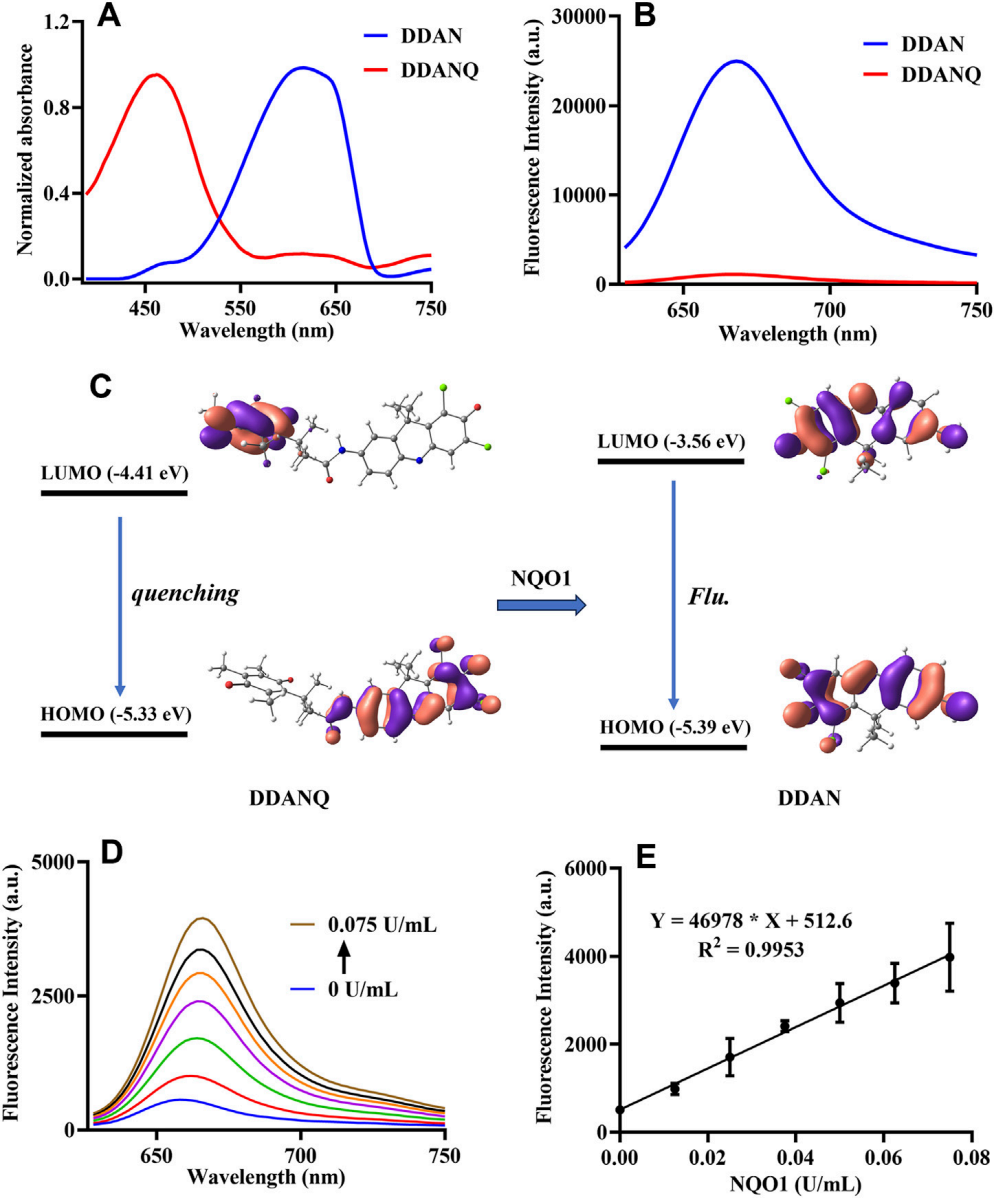
**(A)** The absorption spectra of DDANQ and DDAN. **(B)** The fluorescent spectra of DDANQ and DDAN. **(C)** Calculated HOMO and LUMO molecular orbitals involved in the emission for DDANQ and DDAN. **(D)** Fluorescence spectra of DDANQ incubating with increasing concentrations of NQO1. **(E)** Linear correlation (R^2^ = 0.9953) between fluorescence emission intensity and NQO1 concentrations. (*λ*_ex_ = 600 nm, *λ*_em_ = 666 nm).

Then, the optical properties of DDANQ toward NQO1 were further investigated, as can be seen in Figures 1A,B DDANQ showed shorter absorption and weak fluorescence signals, while the fluorescence signal increased significantly at 666 nm.

The molecular orbital calculation results in Figure 1C showed that the HOMO and LUMO energy difference of DDAN is 1.83 eV. The electrons are mainly distributed in the two benzene rings, which showed the characteristics of local excitation. Compared with DDAN, the energy difference of DDANQ is reduced to 0.92 eV. At the same time, the electron distribution of DDANQ changes obviously, from the fluorophore to the benzoquinone, indicating that it was a charge transfer state, and the pronounced photoinduced electron transfer (PeT) process resulted in fluorescence quenching of the DDANQ molecule.

When DDANQ was incubated with different concentrations of NQO1, the fluorescence emission intensity at 666 nm was increased (Figures 1D,E), demonstrating dose-dependent behavior proportional to NQO1 enzyme concentrations over the 0–0.075 U/mL range, the quantitative equation was Y = 469,78X + 512.6, where Y means fluorescence intensity at 666 nm and X means the concentration of NQO1.

In addition, the stability of DDANQ toward common amino acids and metal ions was also tested, as shown in Supplementary Figure S7, DDANQ showed good stability in Try, Ser, Gln, Gly, Arg, Lys, Glu, Cys, Tyr, Myristic acid, K^+^, Na^+^, Mg^2+^, Ca^2+^, Ba^2+^, Zn^2+^, Cu^2+^, Ni^2+^, Sn^4+^, Mn^2+^, CO_3_^2-^, SO_4_^2-^, Fe^2+^, Fe^3+^, Cl^−^, NO_2_^−^, HPO_4_^2-^, and H_2_PO_4_^−^. These results establish DDANQ as a promising NIR fluorescent biosensor for quantitative determination of NQO1 activity in biological system, demonstrating significant potential for enzyme activity profiling.

### 3.3 The selectivity of DDANQ toward NQO1 and docking simulation

**FIGURE 2:**
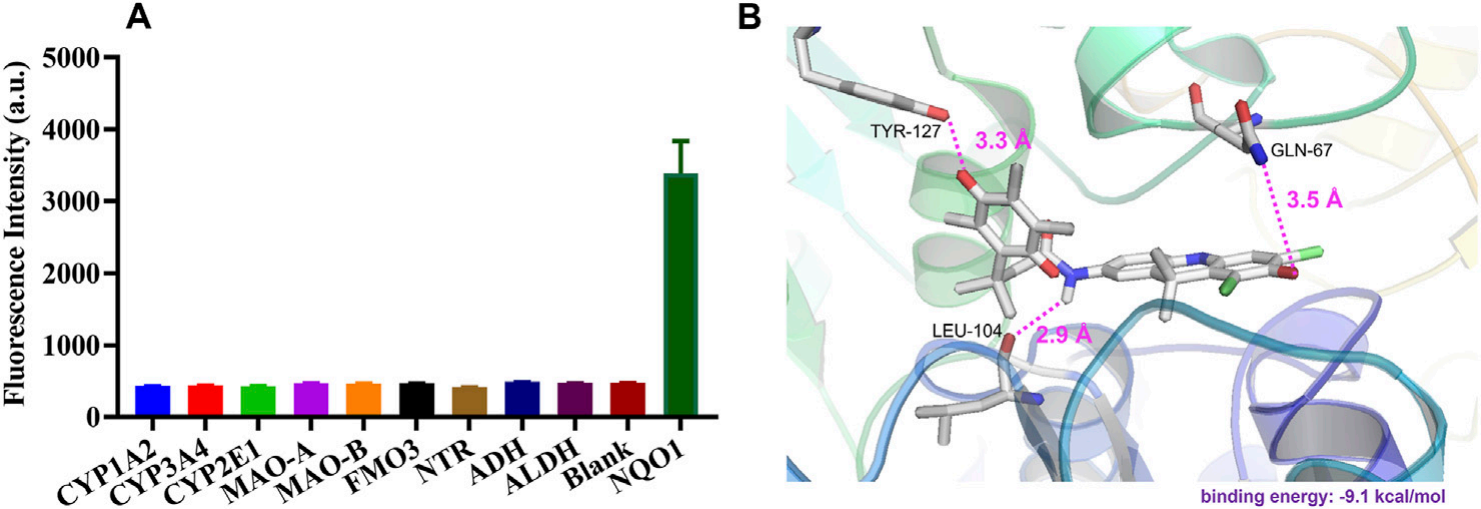
**(A)** Fluorescence response of DDANQ incubation with various oxidoreductase. **(B)** Docking analysis for DDANQ and NQO1 protein. Detailed interaction with the residues that make important contributions to DDANQ binding (*λ*_ex_ = 600 nm, *λ*_em_ = 666 nm).

The specificity of DDANQ toward various oxidoreductase was shown in Figure 2A, only NQO1 triggered a remarkable fluorescence enhancement at 666 nm, while other enzymes showed weak effect on the fluorescence signal. These results indicated that DDANQ was applicable to detect NQO1 activity in complex systems. Next, the interaction mechanism between probe DDANQ and the NOQ1 protein was investigated. The docking simulation revealed a binding energy of −9.1 kcal/mol, indicating a strong and spontaneous binding affinity of probe DDANQ to the NOQ1 protein. For further visualization, PYMOL was employed to analyze the binding interactions (Figure 2B). The results showed that probe DDANQ formed multiple hydrogen bonds with NOQ1. The carbonyl group of the fluorophore matrix acted as a hydrogen bond acceptor, forming a hydrogen bond with the amide hydrogen of GLN-67 (glutamine at position 67), and the bond length was 3.5 Å. The carbonyl group of the diphenyl quinone moiety interacts with the phenolic hydroxyl group of TYR-127 (tyrosine at position 127) *via* a hydrogen bond, with a bond-length of 3.3 Å. Additionally, the amide hydrogen of the probe serves as a proton donor, forming a hydrogen bond with the carbonyl group of LEU-104 (leucine at position 104), with a bond length of 2.9 Å. Moreover, PRO-69 and PRO-103 are positioned above and below the fluorophore matrix, respectively, and cooperate with PHE-18 and ILE-193 to form a hydrophobic pocket around the probe. This hydrophobic interaction enhances the binding affinity of probe DDANQ to NOQ1, facilitating the enzymatic reaction.

### 3.4 Fluorescence analysis of endogenous NQO1 activity in cells and tissues

**FIGURE 3:**
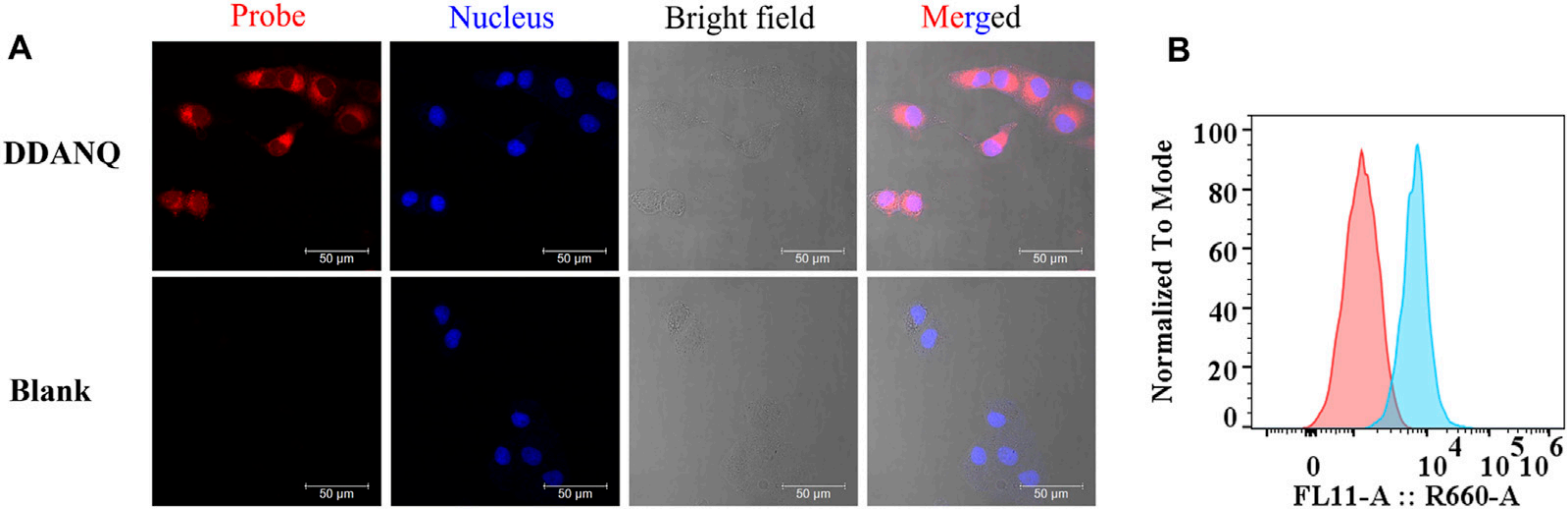
**(A)** Fluorescence analysis of endogenous NQO1 activity of A549 cells by DDANQ. “Blank” means cells without probe. *λ*_ex_ = 633 nm, *λ*_em_ = 645–690 nm. Scale bar is 50 μm. **(B)** Flow cytometric analysis of NQO1 in A549 cells (Red: the blank cells, blue: cells incubated with DDANQ).

**FIGURE 4:**
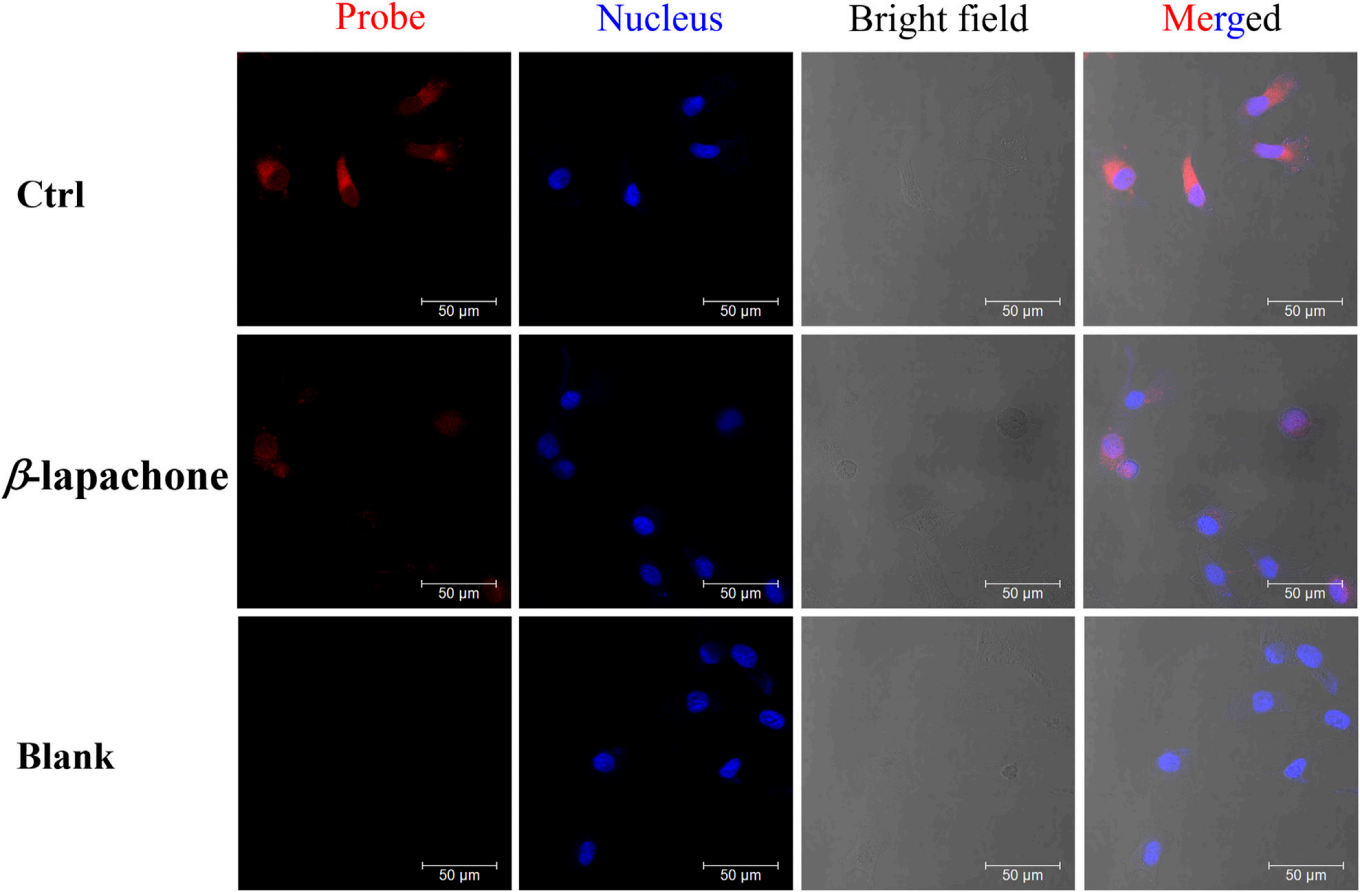
The fluorescence images of NQO1 activity in living MDA-MB-231 cells using DDANQ in presence/ absence of NQO1 inhibitor *β*-lapachone. “Ctrl” means cells with probe, “Blank” means cells without probe. *λ*_ex_ = 633 nm, *λ*_em_ = 645–690 nm. Scale bar is 50 μm.

Encouraged by the results of above experiments results, DDANQ was further used to image endogenous NQO1 in living cells and tissues. DDANQ showed almost no cytotoxicity in A549 cells up to 20 μM by CCK-8 assay (Supplementary Figure S8), which means that the probe has good biosafety in living cells. As Figure 3 showed, A549 cells displayed no fluorescence signal, after the addition of DDANQ, the significant fluorescence signal was achieved, the same phenomenon was observed in Hela cells (Supplementary Figure S9). DDANQ displayed strong fluorescence signal in A549 cells, Hela cells and MDA-MB-231 cells (Figure 4), and was further verified in flow cytometry experiments. Compared with the control group, cancer cells showed varying degrees of displacement after adding DDANQ.

While MDA-MB-231 cells were pre-incubated with NQO1 inhibitor (*β*-lapachone), a weaker fluorescence signal was observed when the probe was added. DDANQ displayed strong fluorescence signal in A549 cells, Hela cells and MDA-MB-231 cells. Collectively, these findings demonstrated that DDANQ has good cell permeability and biosafety, and DDANQ is suitable for the detection of endogenous NQO1 activity with good selectivity in various complex and living bio-systems.

**FIGURE 5:**
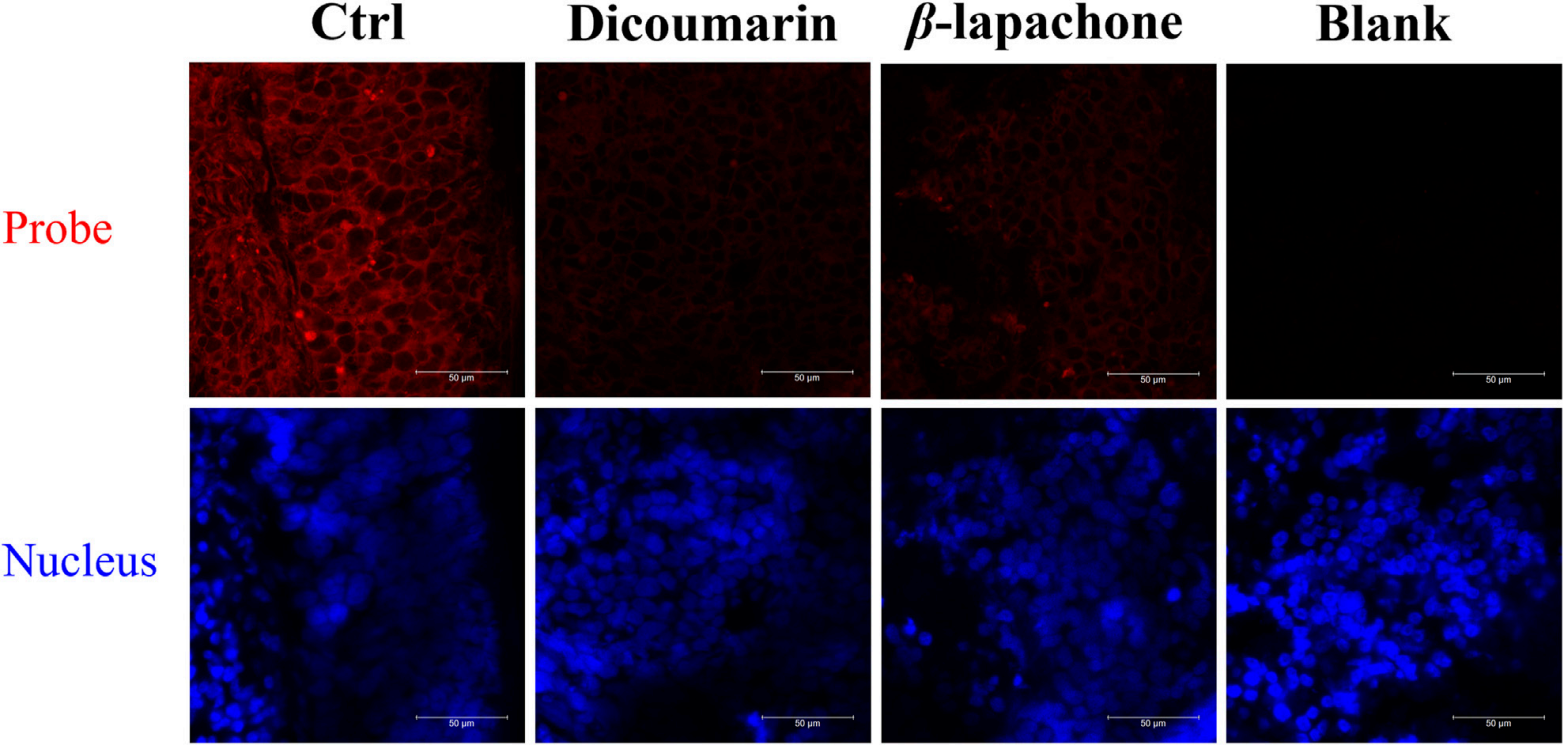
The fluorescence images of NQO1 activity in Hela tumor slices using DDANQ in presence/ absence of NQO1 inhibitor dicoumarin or *β*-lapachone. “Ctrl” means tumor slices with probe, “Blank” means tumor slices without probe. *λ*_ex_ = 633 nm, *λ*_em_ = 645–690 nm. Scale bar is 50 μm.

**FIGURE 6:**
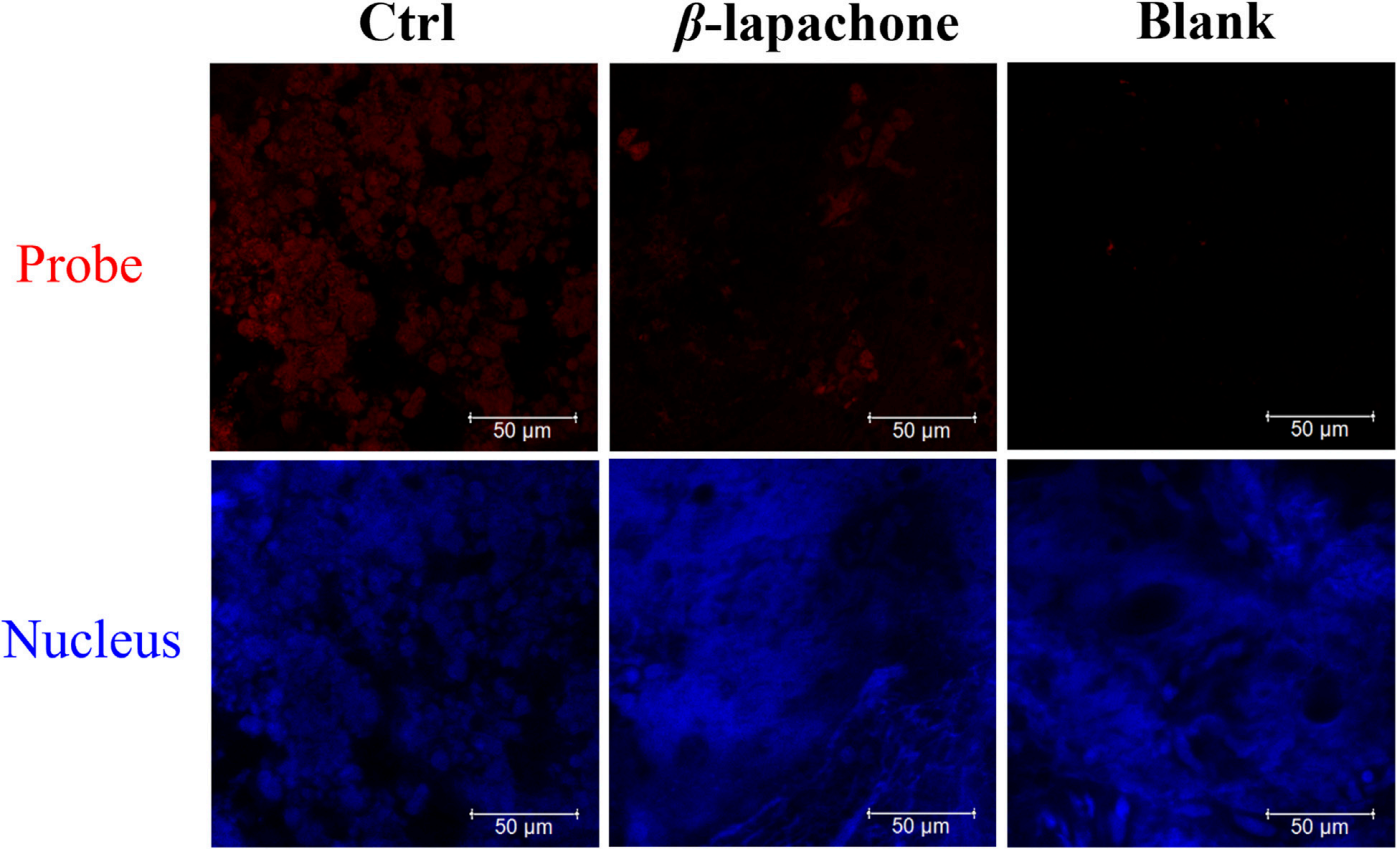
The fluorescence images of NQO1 activity in human breast cancer slices using DDANQ in presence/ absence of NQO1 inhibitor *β*-lapachone. “Ctrl” means samples with probe, “Blank” means samples without probe. *λ*_ex_ = 633 nm, *λ*_em_ = 645–690 nm. Scale bar is 50 μm.

Then, DDANQ was used to detect NQO1 activity in Hela tumor slices and human breast cancer slices. As can be seen in Figure 5, significant NIR fluorescence signals were found when DDANQ was dropped into slices, and the fluorescence signal could be oppressed by adding the NQO1 inhibitors (dicoumarin and *β*-lapachone). The same results were also observed in human breast cancer slices imaging experiment (Figure 6), and the fluorescence intensity of DDANQ produced by NQO1 metabolism decreased significantly after the addition of inhibitor *β*-lapachone. These experimental results suggest that DDANQ has ultra-high sensitivity to detect NQO1 activity in biological samples, may be used as a molecular tool for fluorescent-induced surgical navigation.

## 4 Discussion

NQO1 is a critical cytosolic flavoenzyme that catalyzes the two-electron reduction of quinones to hydroquinones. The elevated NQO1 expression in various cancers correlates with tumor progression and chemotherapeutic resistance (Glorieux et al., 2016), which has been exploited for developing tumor-selective prodrugs (Qu et al., 2020; Yang et al., 2022) and molecular imaging agents (Peng et al., 2022; Khan et al., 2024). In our present work, by strategically integrating benzoquinone as the NQO1 recognition moiety with the fluorophores DDAO and DDAN, we establish a rational design approach for targeted probe construction. Particularly noteworthy is DDANQ’s capability in application across multiple tumor models (Hela, A549, and MDA-MB-231 cells) and clinical specimens. Our findings not only confirm DDANQ’s utility as a research tool for elucidating NQO1’s pathophysiological roles but also suggest its clinical applicability as a precision-guided surgical aid.

## 5 Conclusion

In summary, a NIR fluorescent probe designated as DDANQ was rationally designed and synthesized through a systematic approach. DDAO and DDAN that have excellent optical properties are selected as fluorophores, and according to the metabolic characteristics of NQO1, benzoquinone is introduced as the recognition site to construct fluorescent probes DDAOQ and DDANQ. After screening, DDANQ showed excellent stability, and displayed good selectivity and sensitivity toward NQO1, an important quinone oxidoreductase that displayed chemical protection and biological activation in many physiological and pathological processes, and was highly expressed in many tumors. The optical properties and enzyme selectivity of DDANQ were also elucidated by chemical calculation and molecular docking methods. DDANQ could detect endogenous NQO1 activity in many tumor cells including Hela, A549, and MDA-MB-231 cells, and it could also be used to detect the activity of NQO1 in Hela tumor slices and human breast cancer slices. It was observed that the fluorescence turn-on of DDANQ was strongly suppressed in living cells and tissues, which confirmed the NQO1 inhibitory effects of *β*-lapachone in the complex bio-samples. DDANQ exhibited the excellent NQO1 specificity and imaging capability, was used as a practical molecular tool to study the physiological function of NQO1 and is expected to be a clinical fluorescence-guided tumor resection reagent.

## Data Availability

The original contributions presented in the study are included in the article/Supplementary Material, further inquiries can be directed to the corresponding authors.
