# Supplementary material for: Rational design of a NIR fluorescent probe for highly selective sensing of NAD(P)H quinone oxidoreductase 1 in living systems
Source: Front Chem. 2025 Jun 24;13:1626741. doi: 10.3389/fchem.2025.1626741 (PMC12235910; doi:10.3389/fchem.2025.1626741)
Supplement: Supplementary file 1 [file DataSheet1.pdf]

Supplementary Material

Rational Design of A NIR Fluorescent Probe for Highly Selective Sensing of NAD(P)H quinone oxidoreductase 1 in Living Systems

Fangyu Yang[1†], Li Zhu[2†], Fei Yan[1,2*], Houli Zhang[3*], and Guobiao Liang[1*]

[1]General Hospital of Northern Theater Command, Department of Neurosurgery, Shenyang, China

[2]Second Affiliated Hospital, Dalian Medical University, Dalian, China

[3]College of pharmacy, Dalian Medical University, Dalian, China

*** Correspondence:**
Fei Yan
yfyanf@163.com

Houli Zhang

houlizh@163.com

Guobiao Liang

liangguobiao6708@vip.163.com

[†]These authors have contributed equally to this work

Table of Contents

Supplementary Scheme S1. Synthetic route of **DDAOQ**.. 3

Supplementary Scheme S2. Synthetic route of **DDANQ**.. 3

Supplementary Figure S1. ^{1}H NMR of **DDAOQ** ($CDCl_3$). 3

Supplementary Figure S2. ^{13}C NMR of **DDAOQ** ($CDCl_3$). 4

Supplementary Figure S3. HRMS of **DDAOQ** (ESI positive). 4

Supplementary Figure S4. ^{1}H NMR of **DDANQ** ($CDCl_3$)... 5

Supplementary Figure S5. ^{13}C NMR of **DDANQ** ($CDCl_3$). 5

Supplementary Figure S6. HRMS of **DDANQ** (ESI negative).................................... 6

Supplementary Figure S7. Fluorescence response of **DDANQ** incubation with common amino acids and metal ions.. 6

Supplementary Figure S8. Cytotoxicity assay of **DDANQ** in A549 cells..................... 6

Supplementary Figure S9. (A) Fluorescence imaging of NQO1 in Hela cells. “Blank” means cells without probe. Scale bar is 50 μm. (B) Flow cytometric analysis of NQO1 in Hela cells. (Red: the blank cells, blue: cells incubated with **DDANQ**)... 7

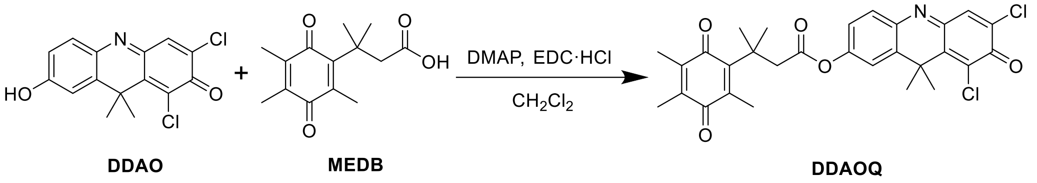

Supplementary Scheme S1. Synthetic route of **DDAOQ**.

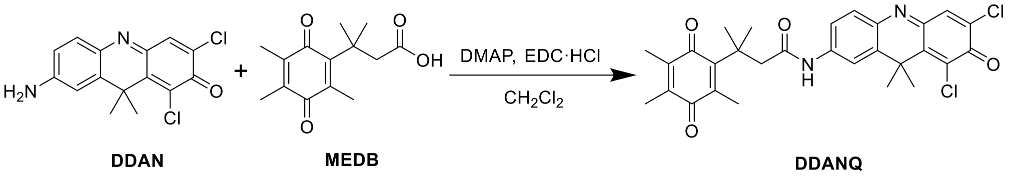

Supplementary Scheme S2. Synthetic route of **DDANQ**.

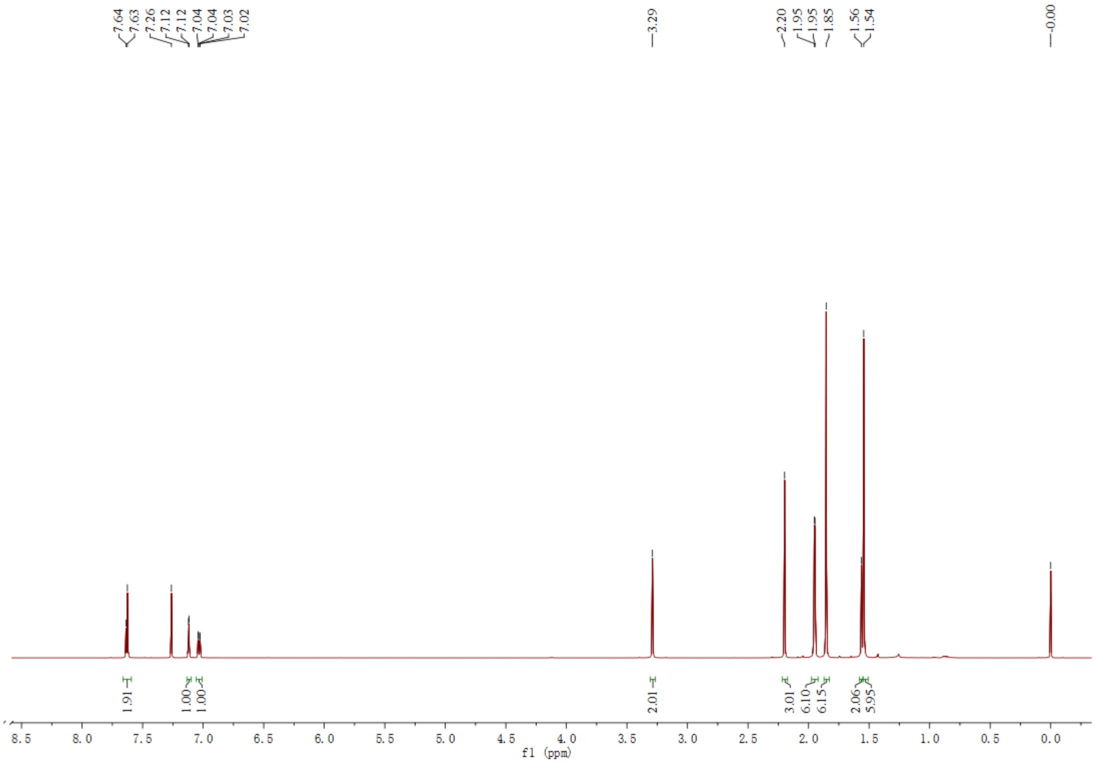

Supplementary Figure S1. ^{1}H NMR of **DDAOQ** ($CDCl_3$).

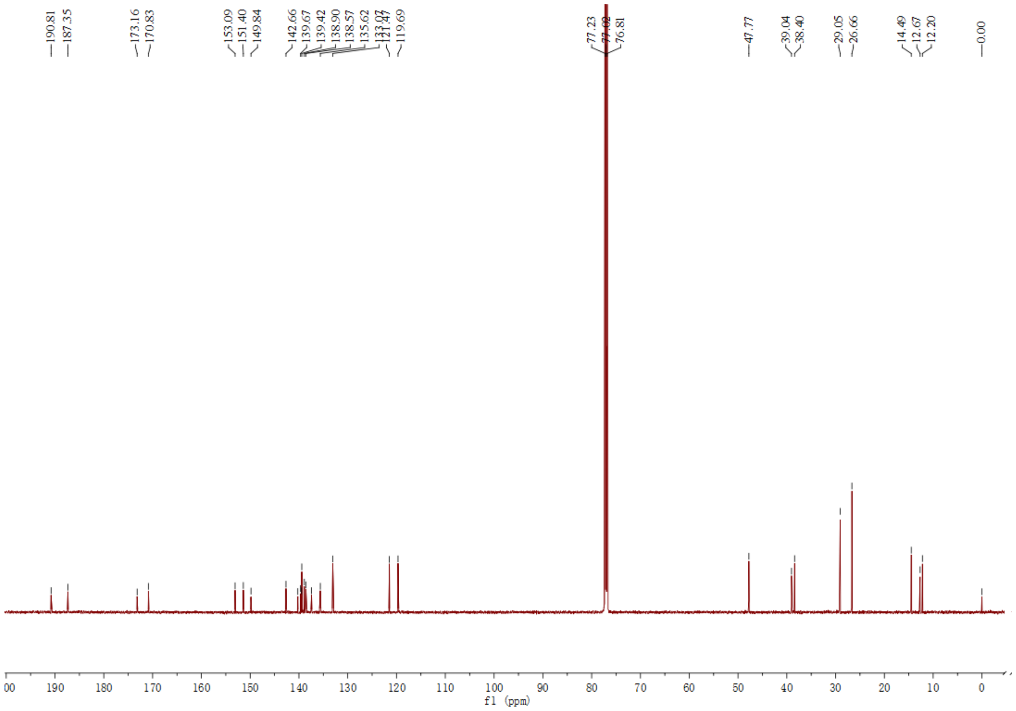

Supplementary Figure S2. ^{13}C NMR of **DDAOQ** ($CDCl_3$).

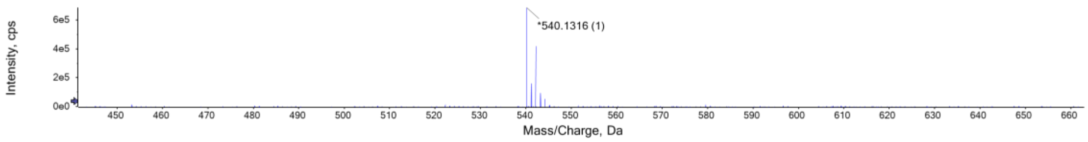

Supplementary Figure S3. HRMS of **DDAOQ** (ESI positive).

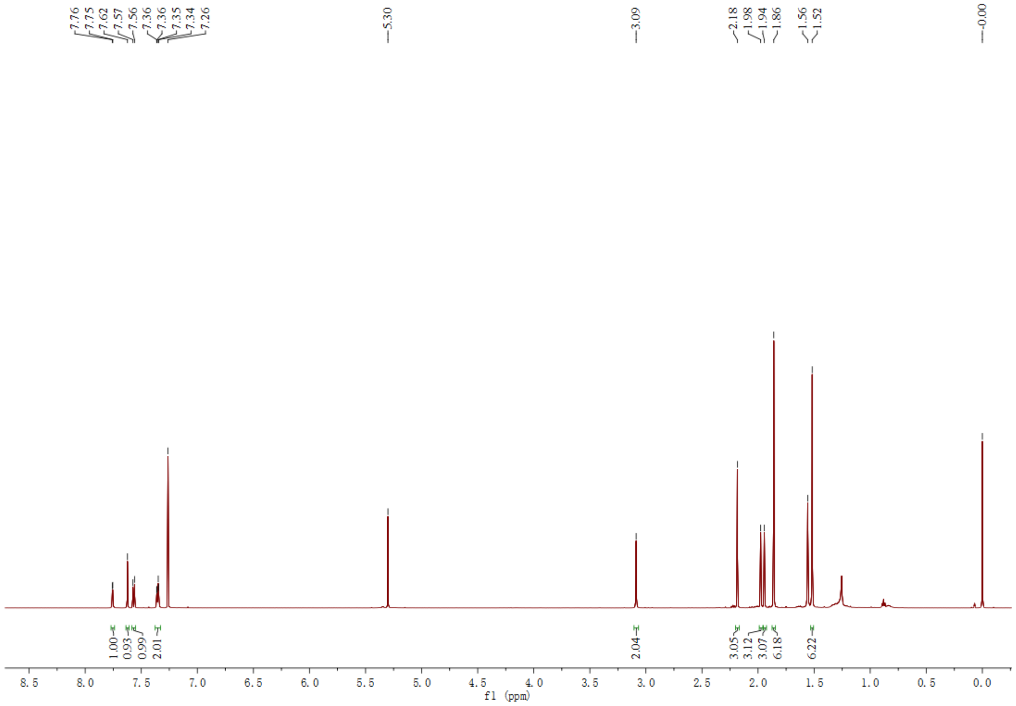

Supplementary Figure S4. ^{1}H NMR of **DDANQ** ($CDCl_3$).

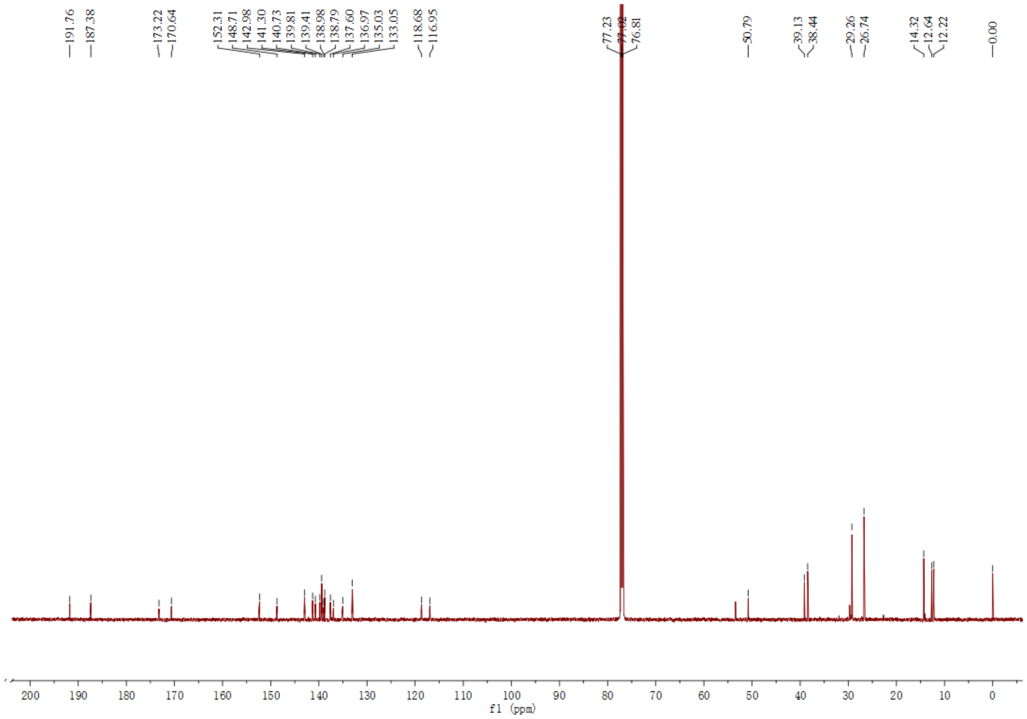

Supplementary Figure S5. ^{13}C NMR of **DDANQ** ($CDCl_3$).

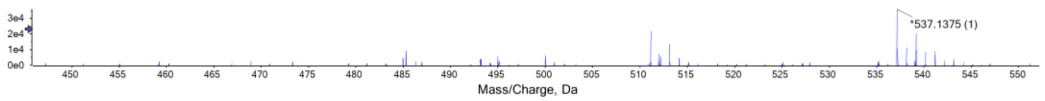

Supplementary Figure S6. HRMS of **DDANQ** (ESI negative).

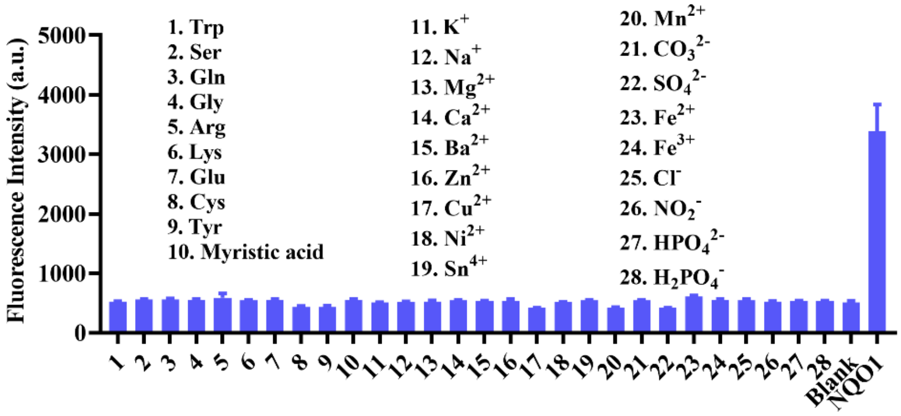

Supplementary Figure S7. Fluorescence response of **DDANQ** incubation with common amino acids and metal ions.

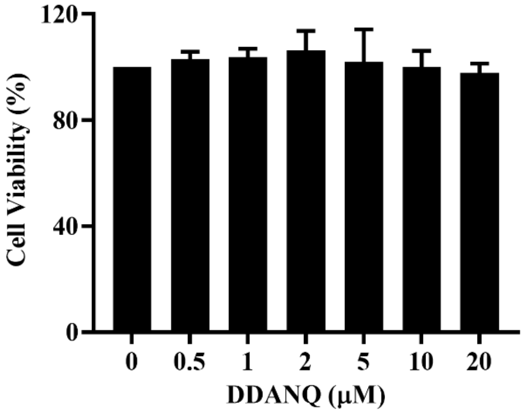

Supplementary Figure S8. Cytotoxicity assay of **DDANQ** in A549 cells.

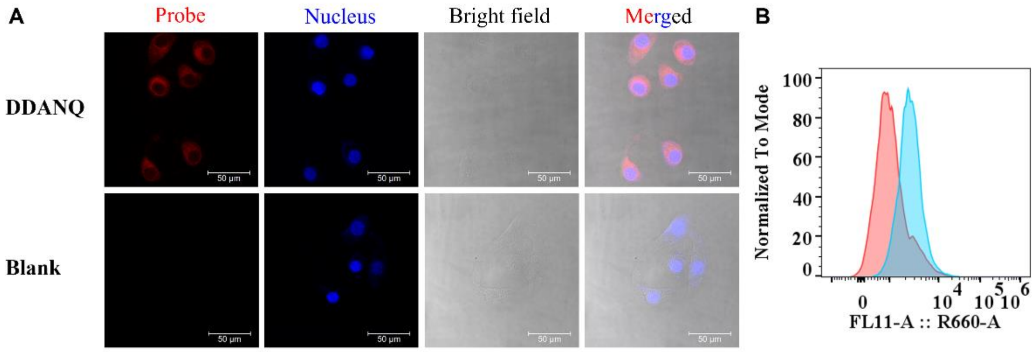

Supplementary Figure S9. (A) Fluorescence imaging of NQO1 in Hela cells. “Blank” means cells without probe. Scale bar is 50 μm. (B) Flow cytometric analysis of NQO1 in Hela cells. (Red: the blank cells, blue: cells incubated with **DDANQ**).
